# Supplementary material for: Effectiveness and Safety of Different Treatment Modalities for Patients Older Than 60 Years with Distal Radius Fracture: A Network Meta-Analysis of Clinical Trials
Source: Int J Environ Res Public Health. 2023 Feb 19;20(4):3697. doi: 10.3390/ijerph20043697 (PMC9965012; doi:10.3390/ijerph20043697)
Supplement: Supplementary file 1 [file ijerph-20-03697-s001.zip › Figures S1 to S3. Network plot.pdf]

Figure S1. Contribution plot for the DASH questionnaire.

Direct comparisons in the network

Network meta-analysis estimates	BEFvsNBEF	BEFvsVLP	CIvsVLP	IMNvsVLP	PKWvsVLP
**Mixed estimates**					
BEFvsNBEF	99.9	0.1			
BEFvsVLP	0.4	98.9	0.2	0.2	0.2
CIvsVLP			100.0		
IMNvsVLP	0.5	1.1	0.5	97.3	0.5
PKWvsVLP	0.2	0.4	0.2	0.2	99.1
**Indirect estimates**					
BEFvsCI	0.2	49.7	49.9	0.1	0.1
BEFvsIMN	0.5	49.7	0.2	49.5	0.2
BEFvsPKW	0.3	49.8			49.9
CIvsIMN	0.3	0.5	49.9	49.1	0.3
CIvsPKW	0.1	0.2	50.0	0.1	49.7
CIvsNBEF	33.3	33.2	33.4	0.1	0.1
IMNvsPKW	0.2	0.4	0.2	49.4	49.9
IMNvsNBEF	33.5	33.2	0.1	33.1	0.1
PKWvsNBEF	33.4	33.2			33.3
VLPvsNBEF	49.9	49.7	0.1	0.1	0.1
**Entire network**	18.0	28.5	18.0	17.6	17.9
**Included studies**	1	1	8	1	3

The size of each square is proportional to the weight attached to each direct summary effect (horizontal axis) for the estimation of each network summary effects (vertical axis). The numbers re-express the weight as percentages.
BEF: Bridging external fixation; **CI:** Cast immobilization; **IM:** Intramedullary nail; **NBEF:** Nonbridging external fixation; **PKW:** Percutaneous kirshner wire; **VLP:** Volar locking plate.

Figure S2. Contribution plot for the PRWE questionnaire.

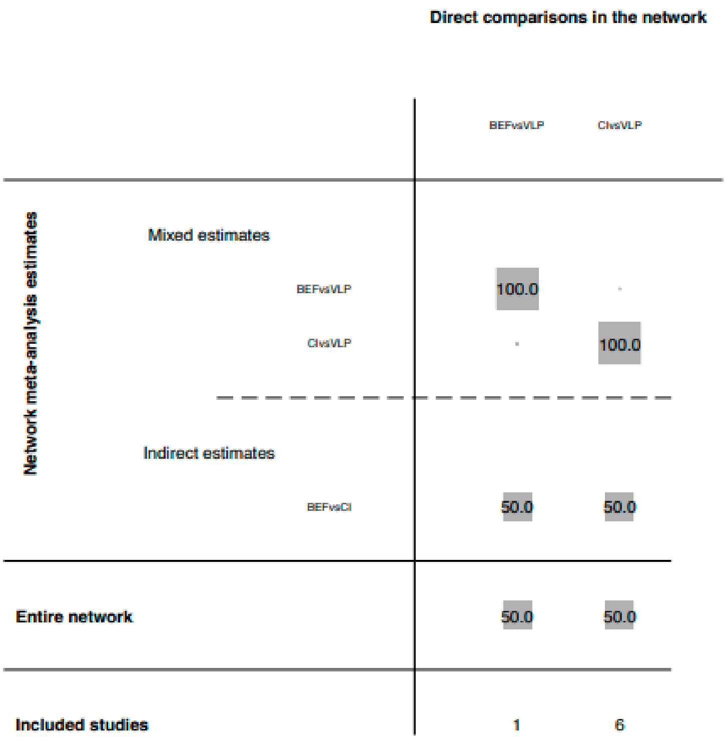

The size of each square is proportional to the weight attached to each direct summary effect (horizontal axis) for the estimation of each network summary effects (vertical axis). The numbers re-express the weight as percentages.
BEF: Bridging external fixation; **CI:** Cast immobilization; **VLP:** Volar locking plate.

Figure S3. Contribution plot for the Grip strength.

Direct comparisons in the network

Network meta-analysis estimates	BEFvsCI	BEFvsPKW	BEFvsVLP	CIvsPKW	CIvsVLP	DPFvsVLP	PKWvsVLP
**Mixed estimates**							
BEFvsCI	21.3	13.4	22.9	19.6	16.6		6.2
BEFvsPKW	18.0	11.3	19.3	32.0	14.0		5.3
BEFvsVLP	13.8	8.6	52.7	2.5	16.3	0.1	6.1
CIvsPKW				99.9			
CIvsVLP	11.6	7.3	18.9	19.1	31.3	0.1	11.8
DPFvsVLP			0.3	0.1	0.3	99.1	0.1
PKWvsVLP	9.4	5.9	15.2	34.7	25.3		9.5
**Indirect estimates**							
BEFvsDPF	7.9	5.0	30.1	1.4	9.2	42.8	3.5
CIvsDPF	7.2	4.5	11.6	11.9	19.3	38.2	7.3
DPFvsPKW	6.3	3.9	10.1	23.2	16.8	33.3	6.4
**Entire network**	10.0	6.2	18.3	21.8	16.2	21.3	6.1
**Included studies**	3	1	3	2	7	1	4

The size of each square is proportional to the weight attached to each direct summary effect (horizontal axis) for the estimation of each network summary effects (vertical axis). The numbers re-express the weight as percentages.

BEF: Bridging external fixation; **CI:** Cast immobilization; **DPF:** Dorsal plate fixation; **IM:** Intramedullary nail; **PKW:** Percutaneous kirshner wire; **VLP:** Volar locking plate.
