# Supplementary material for: Effectiveness and Safety of Different Treatment Modalities for Patients Older Than 60 Years with Distal Radius Fracture: A Network Meta-Analysis of Clinical Trials
Source: Int J Environ Res Public Health. 2023 Feb 19;20(4):3697. doi: 10.3390/ijerph20043697 (PMC9965012; doi:10.3390/ijerph20043697)

Figure S4. Funnel plot for grip strength (volar locking plate versus cast mobilization, (p= 0.79)

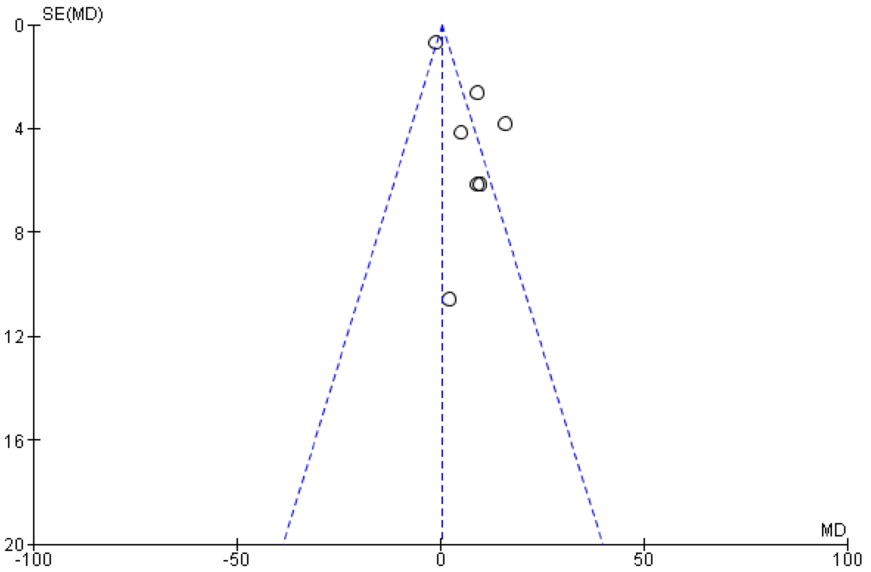

Figure S5. Funnel plot for DASH (volar locking plate versus k-wire fixation (p= 0.82)

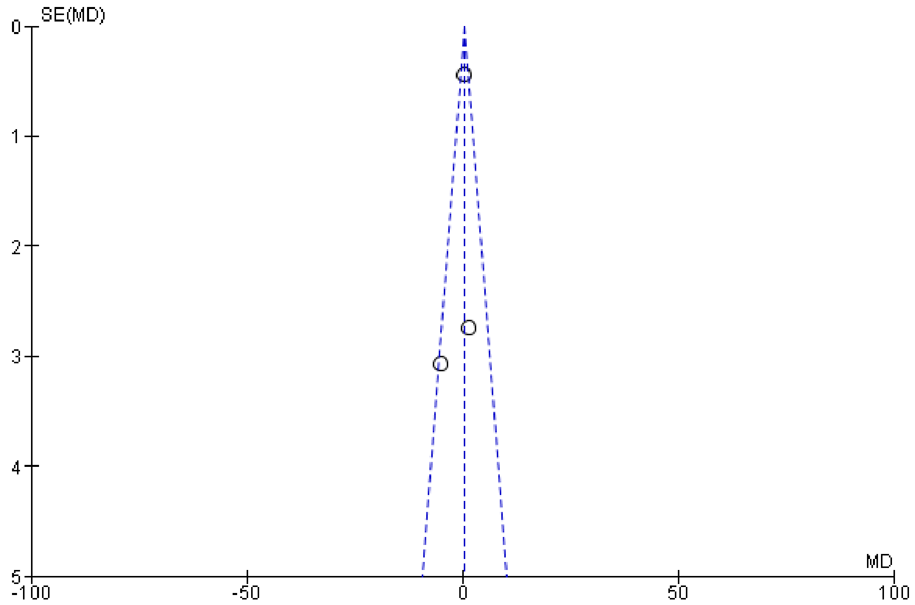

Figure S6. Funnel plot PRWE (volar locking plate versus bridging external fixation, p = 0.68)

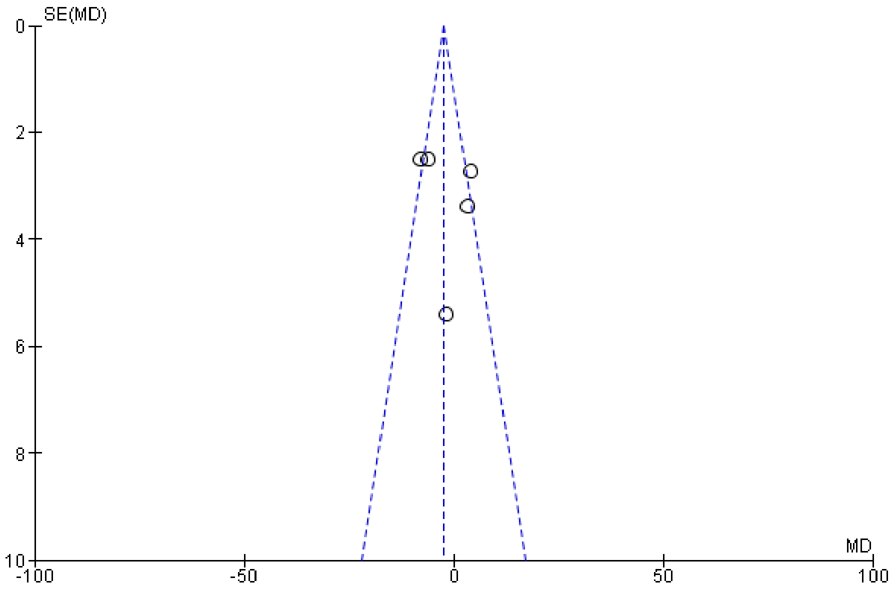

Figure S7. Funnel plot complications (percutaneous K-wire fixation versus cast immobilization, p = 0.85)

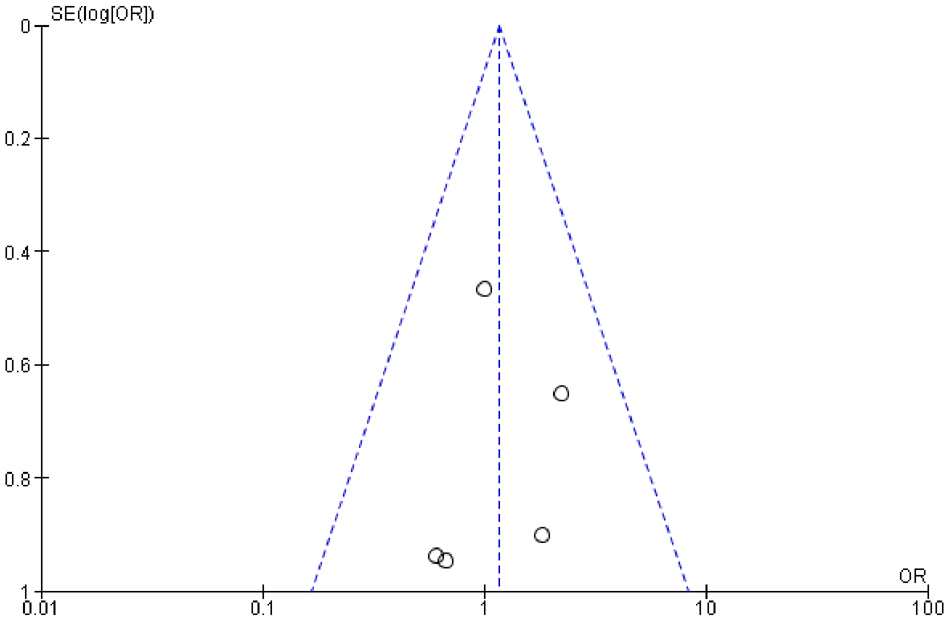

Supplement: Supplementary file 1 [file ijerph-20-03697-s001.zip › Figures S4 to S7. Funnel plot.pdf]
