# Supplementary material for: Effectiveness and Safety of Different Treatment Modalities for Patients Older Than 60 Years with Distal Radius Fracture: A Network Meta-Analysis of Clinical Trials
Source: Int J Environ Res Public Health. 2023 Feb 19;20(4):3697. doi: 10.3390/ijerph20043697 (PMC9965012; doi:10.3390/ijerph20043697)
Supplement: Supplementary file 1 [file ijerph-20-03697-s001.zip › Table S2. Supplemental search strategy.pdf]

Table S2. Searches strategies

Database	Search strategy	Results
**Medline**	((((colles' fracture[MeSH Terms]) OR (radius fracture[MeSH Terms])) OR (distal radius fracture[Text Word])) AND ((((((((((((((((surgical procedures, operative[MeSH Terms]) OR (fracture fixation[MeSH Terms])) OR (fracture fixation, internal[MeSH Terms])) OR (fracture fixation, intramedullary[MeSH Terms])) OR (Closed fracture reduction[MeSH Terms])) OR (Open reduction[Text Word])) OR (Internal fixation[Text Word])) OR (External fixation[Text Word])) OR (Bridging external fixation[Text Word])) OR (Nonbridging external fixation[Text Word])) OR (Kirschner wire fixation[Text Word])) OR (Percutaneous K-wire fixation[Text Word])) OR (Volar locked plating fixation[Text Word])) OR (Volar locking plating system[Text Word])) OR (Closed Fracture Reduction[Text Word])) OR (Closed reduction[Text Word])) OR (Plaster cast[Text Word])) OR (Cast Immobilization[Text Word]))) AND ((((((((Randomized Controlled Trial [pt]) OR Controlled Clinical Trial [pt]) OR Randomized [tiab]) OR Randomly [tiab]) OR Trial) OR Groups [tiab])) NOT ((Animals [mh]) NOT Humans [mh])))	**1286**
**EMBASE**	(colles AND ('fracture'/exp OR fracture) OR (('radius'/exp OR radius) AND ('fracture'/exp OR fracture)) OR (distal AND ('radius'/exp OR radius) AND ('fracture'/exp OR fracture))) AND (surgical AND ('procedures,'/exp OR procedures,) AND operative OR (('fracture'/exp OR fracture) AND ('fixation'/exp OR fixation)) OR (('fracture'/exp OR fracture) AND fixation, AND internal) OR (('fracture'/exp OR fracture) AND fixation, AND intramedullary) OR (open AND ('reduction'/exp OR reduction)) OR (internal AND ('fixation'/exp OR fixation)) OR (external AND ('fixation'/exp OR fixation)) OR (bridging AND external AND ('fixation'/exp OR fixation)) OR (nonbridging AND external AND ('fixation'/exp OR fixation)) OR (kirschner AND ('wire'/exp OR wire) AND ('fixation'/exp OR fixation)) OR (percutaneous AND ('k wire'/exp OR 'k wire') AND ('fixation'/exp OR fixation)) OR (volar AND locked AND plating AND ('fixation'/exp OR fixation)) OR (volar AND locking AND plating AND system) OR (closed AND ('fracture'/exp OR fracture) AND ('reduction'/exp OR reduction)) OR (closed AND ('reduction'/exp OR reduction)) OR (('plaster'/exp OR plaster) AND ('cast'/exp OR cast)) OR (('cast'/exp OR cast) AND ('immobilization'/exp OR immobilization))) AND (randomized AND controlled AND ('trial'/exp OR trial) OR (controlled AND ('clinical'/exp OR clinical) AND ('trial'/exp OR trial))	**1805**

	OR randomized OR randomly OR 'trial'/exp<br>OR trial OR groups)	
**CENTRAL**	(((colles' fracture) OR (radius fracture)) OR (distal radius fracture)):ti,ab,kw AND (((((((((((((((((surgical procedures, operative) OR (fracture fixation)) OR (fracture fixation, internal)) OR (fracture fixation, intramedullary)) OR (Closed fracture reduction)) OR (Open reduction)) OR (Internal fixation)) OR (External fixation)) OR (Bridging external fixation)) OR (Nonbridging external fixation)) OR (Kirschner wire fixation)) OR (Percutaneous K-wire fixation)) OR (Volar locked plating fixation)) OR (Volar locking plating system)) OR (Closed Fracture Reduction)) OR (Closed reduction)) OR (Plaster cast)) OR (Cast Immobilization)):ti,ab,kw<br><br>Limits: Content type: Trials	**789**
**LILACS**	(((((((((((((((((surgical procedures, operative) OR (fracture fixation)) OR (fracture fixation, internal)) OR (fracture fixation, intramedullary)) OR (closed fracture reduction)) OR (open reduction)) OR (internal fixation)) OR (external fixation)) OR (bridging external fixation)) OR (nonbridging external fixation)) OR (kirschner wire fixation)) OR (percutaneous k-wire fixation)) OR (volar locked plating fixation)) OR (volar locking plating system)) OR (closed fracture reduction)) OR (closed reduction)) OR (plaster cast)) OR (cast immobilization)) AND ((((((randomized controlled trial) OR (controlled clinical trial)) OR (randomized)) OR (randomly)) OR (trial)) OR (groups)) AND (((colles' fracture) OR (radius fracture)) OR (distal radius fracture)) AND (db:("LILACS"))	**34**
**CINHAL**	(((colles' fracture) OR (radius fracture)) OR (distal radius fracture)) AND (((((((((((((((((surgical procedures, operative) OR (fracture fixation)) OR (fracture fixation, internal)) OR (fracture fixation, intramedullary)) OR (Closed fracture reduction)) OR (Open reduction)) OR (Internal fixation)) OR (External fixation)) OR (Bridging external fixation)) OR (Nonbridging external fixation)) OR (Kirschner wire fixation)) OR (Percutaneous K-wire fixation)) OR (Volar locked plating fixation)) OR (Volar locking plating system)) OR (Closed Fracture Reduction)) OR (Closed reduction)) OR (Plaster cast)) OR (Cast Immobilization)) AND ((((((Randomized Controlled Trial) OR (Controlled Clinical Trial)) OR (Randomized)) OR (Randomly)) OR (Trial)) OR (Groups))	**536**
**SPORTDISCUS**	(((colles' fracture) OR (radius fracture)) OR (distal radius fracture)) AND (((((((((((((((((surgical procedures, operative) OR (fracture fixation)) OR (fracture fixation, internal)) OR (fracture fixation, intramedullary)) OR (Closed fracture reduction)) OR (Open reduction)) OR (Internal fixation)) OR (External fixation)) OR (Bridging external fixation)) OR (Nonbridging external fixation)) OR (Kirschner wire fixation)) OR (Percutaneous K-wire fixation)) OR (Volar locked plating	**133**

	fixation)) OR (Volar locking plating system)) OR (Closed Fracture Reduction)) OR (Closed reduction)) OR (Plaster cast)) OR (Cast Immobilization)) AND ((((((Randomized Controlled Trial) OR (Controlled Clinical Trial)) OR (Randomized)) OR (Randomly)) OR (Trial)) OR (Groups))	
**Web of science**	((ALL=(((colles' fracture) OR (radius fracture)) OR (distal radius fracture))) AND ALL=((((((Randomized Controlled Trial) OR (Controlled Clinical Trial)) OR (Randomized)) OR (Randomly)) OR (Trial)) OR (Groups))) AND ALL=(((((((((((((((((surgical procedures, operative) OR (fracture fixation)) OR (fracture fixation, internal)) OR (fracture fixation, intramedullary)) OR (Closed fracture reduction)) OR (Open reduction)) OR (Internal fixation)) OR (External fixation)) OR (Bridging external fixation)) OR (Nonbridging external fixation)) OR (Kirschner wire fixation)) OR (Percutaneous K-wire fixation)) OR (Volar locked plating fixation)) OR (Volar locking plating system)) OR (Closed Fracture Reduction)) OR (Closed reduction)) OR (Plaster cast)) OR (Cast Immobilization))	**1462**
**PEDro**	Abstract & title: Radius fracture<br>Body part: Hand or Wrist<br>Method: Clinical trial	**52**
**Total**		**6097**

* All searches were carried out on November 30, 2022.
