# Supplementary material for: Effectiveness and Safety of Different Treatment Modalities for Patients Older Than 60 Years with Distal Radius Fracture: A Network Meta-Analysis of Clinical Trials
Source: Int J Environ Res Public Health. 2023 Feb 19;20(4):3697. doi: 10.3390/ijerph20043697 (PMC9965012; doi:10.3390/ijerph20043697)
Supplement: Supplementary file 1 [file ijerph-20-03697-s001.zip › Table S3. Excluded studies.pdf]

Table S3. Excluded studies

Study (First author/year)	Reference	Reason for exclusion
Abbaszadegan et al 1990	External fixation or plaster cast for severely displaced Colles' fractures? Prospective 1-year study of 46 patients. Acta Orthop Scand. 1990 Dec;61(6):528-30.	Patients < 60 years
Abramo et al 2009	Open reduction and internal fixation compared to closed reduction and external fixation in distal radial fractures: a randomized study of 50 patients. Acta Orthop. 2009 Aug;80(4):478-85.	Patients < 60 years
Aita et al 2014	Randomized clinical trial on percutaneous minimally invasive osteosynthesis of fractures of the distal extremity of the radius. Rev Bras Ortop. 2014 Apr 18;49(3):218-26.	Patients < 60 years
Aktekin et al 2010	Comparison between external fixation and cast treatment in the management of distal radius fractures in patients aged 65 years and older. J Hand Surg Am. 2010 May;35(5):736-42.	Nonrandomized controlled trial
Andrade-Silva et al 2019	Influence of postoperative immobilization on pain control of patients with distal radius fracture treated with volar locked plating: A prospective, randomized clinical trial. Injury. 2019 Feb;50(2):386-391.	Other comparison
Andreasson et al 2020	Functional outcome after corrective osteotomy for malunion of the distal radius: a randomised, controlled, double-blind trial. Int Orthop. 2020 Jul;44(7):1353-1365.	Other comparison
Arora et al 2009	A comparative study of clinical and radiologic outcomes of unstable colles type distal radius fractures in patients older than 70 years: nonoperative treatment versus volar locking plating. J Orthop Trauma. 2009 Apr;23(4):237-42.	Nonrandomized controlled trial
Athar et al 2018	Is external fixation a better way than plaster to supplement K-wires in non-comminuted distal radius fractures? Postgrad Med J. 2018 Jan;94(1107):20-24.	Patients < 60 years
Bahari-Kashani et al 2013	Outcomes of pin and plaster versus locking plate in distal radius intraarticular fractures. Trauma Mon. 2013 Winter;17(4):380-5.	Patients < 60 years
Cassidy et al 2003	Norian SRS cement compared with conventional fixation in distal radial fractures. A randomized study. J Bone Joint Surg Am. 2003 Nov;85(11):2127-37.	Patients < 60 years
Chung et al 2008	Comparative outcomes study using the volar locking plating system for distal radius fractures in both young adults and adults older than 60 years. J Hand Surg Am. 2008 Jul-Aug;33(6):809-19.	Other comparison

Costa et al 2014	Percutaneous fixation with Kirschner wires versus volar locking plate fixation in adults with dorsally displaced fracture of distal radius: randomised controlled trial. BMJ. 2014 Aug 5;349:g4807.	Patients < 60 years
Costa et al 2019	Percutaneous fixation with Kirschner wires versus volar locking-plate fixation in adults with dorsally displaced fracture of distal radius: five-year follow-up of a randomized controlled trial. Bone Joint J. 2019 Aug;101-B(8):978-983.	Patients < 60 years
Costa et al 2022	Surgical fixation with K-wires versus casting in adults with fracture of distal radius: DRAFFT2 multicentre randomised clinical trial. BMJ. 2022 Jan 19;376:e068041.	Patients < 60 years
Drovetz et al 2016	Volar locking distal radius plates show better short-term results than other treatment options: A prospective randomised controlled trial. World J Orthop. 2016 Oct 18;7(10):687-694.	Patients < 60 years
Egol et al 2008	Bridging external fixation and supplementary Kirschner-wire fixation versus volar locked plating for unstable fractures of the distal radius: a randomised, prospective trial. J Bone Joint Surg Br. 2008 Sep;90(9):1214-21.	Patients < 60 years
Egol et al 2010	Distal radial fractures in the elderly: operative compared with nonoperative treatment. J Bone Joint Surg Am. 2010 Aug 4;92(9):1851-7.	Nonrandomized controlled trial
Figl et al 2009	Volar fixed-angle plate osteosynthesis of unstable distal radius fractures: 12 months results. Arch Orthop Trauma Surg. 2009 May;129(5):661-9.	Nonrandomized controlled trial
Figl 2010	Unstable distal radius fractures in the elderly patient--volar fixed-angle plate osteosynthesis prevents secondary loss of reduction. J Trauma. 2010 Apr;68(4):992-8.	Nonrandomized controlled trial
Földhazy et al 2007	Long-term outcome of nonsurgically treated distal radius fractures. J Hand Surg Am. 2007 Nov;32(9):1374-84.	Patients < 60 years
Franck et al 2000	Distal radius fracture--is non-bridging articular external fixator a therapeutic alternative? A prospective randomized study. Unfallchirurg. 2000 Oct;103(10):826-33.	Patients < 60 years
Gradl et al 2013	Non-bridging external fixation employing multiplanar K-wires versus volar locked plating for dorsally displaced fractures of the distal radius. Arch Orthop Trauma Surg. 2013 May;133(5):595-602.	Patients < 60 years
Gradl et al 2014	Intramedullary nail versus volar plate fixation of extra-articular distal radius fractures. Two-year results of a prospective randomized trial. Injury. 2014 Jan;45 Suppl 1:S3-8.	Patients < 60 years
Gradl et al 2016	Fixation of intra-articular fractures of the distal radius using intramedullary nailing: a randomized trial versus palmar locking plates. Injury. 2016 Dec;47 Suppl 7:S25-S30.	Patients < 60 years

Grewal et al 2005	A randomized prospective study on the treatment of intra-articular distal radius fractures: open reduction and internal fixation with dorsal plating versus mini open reduction, percutaneous fixation, and external fixation. J Hand Surg Am. 2005 Jul;30(4):764-72.	Patients < 60 years
Grewal et al 2011	Open reduction internal fixation versus percutaneous pinning with external fixation of distal radius fractures: a prospective, randomized clinical trial. J Hand Surg Am. 2011 Dec;36(12):1899-906.	Patients < 60 years
Gruber et al 2008	Volar plate fixation of AO type C2 and C3 distal radius fractures, a single-center study of 55 patients. J Orthop Trauma. 2008 Aug;22(7):467-72.	Nonrandomized controlled trial
Günay et al 2015	Which modality is the best choice in distal radius fractures treated with two different Kirschner wire fixation and immobilization techniques? Ulus Travma Acil Cerrahi Derg. 2015 Mar;21(2):119-26.	Patients < 60 years
Hammer et al 2019	Volar Locking Plates Versus Augmented External Fixation of Intra-Articular Distal Radial Fractures: Functional Results from a Randomized Controlled Trial. J Bone Joint Surg Am. 2019 Feb 20;101(4):311-321.	Patients < 60 years
Harley et al 2004	Augmented external fixation versus percutaneous pinning and casting for unstable fractures of the distal radius--a prospective randomized trial. J Hand Surg Am. 2004 Sep;29(5):815-24.	Patients < 60 years
Hayes et al 2008	Bridging and non-bridging external fixation in the treatment of unstable fractures of the distal radius: a retrospective study of 588 patients. Acta Orthop. 2008 Aug;79(4):540-7.	Nonrandomized controlled trial
Howard et al 1989	External fixation or plaster for severely displaced comminuted Colles' fractures? A prospective study of anatomical and functional results. J Bone Joint Surg Br. 1989 Jan;71(1):68-73.	Patients < 60 years
Kapoor et al 2000	Displaced intra-articular fractures of distal radius: a comparative evaluation of results following closed reduction, external fixation and open reduction with internal fixation. Injury. 2000 Mar;31(2):75-9.	Patients < 60 years
Karantana et al 2013	Surgical treatment of distal radial fractures with a volar locking plate versus conventional percutaneous methods: a randomized controlled trial. J Bone Joint Surg Am. 2013 Oct 2;95(19):1737-44.	Patients < 60 years
Kim et al 2021	The Effect of Providing Audiovisual Surgical Information on Decisional Conflict in Patients Undergoing Plate Fixation for Distal Radius Fractures. Clin Orthop Surg. 2021 Mar;13(1):18-23.	Other comparison

Kreder et al 2005	Indirect reduction and percutaneous fixation versus open reduction and internal fixation for displaced intra-articular fractures of the distal radius: a randomised, controlled trial. J Bone Joint Surg Br. 2005 Jun;87(6):829-36.	Patients < 60 years
Krishnan et al 2003	Intra-articular fractures of the distal radius: a prospective randomised controlled trial comparing static bridging and dynamic non-bridging external fixation. J Hand Surg Br. 2003 Oct;28(5):417-21.	Patients < 60 years
Krukhaug et al 2009	External fixation of fractures of the distal radius: a randomized comparison of the Hoffman compact II non-bridging fixator and the Dynawrist fixator in 75 patients followed for 1 year. Acta Orthop. 2009 Feb;80(1):104-8.	Patients < 60 years
Landgren et al 2011	External or internal fixation in the treatment of non-reducible distal radial fractures? Acta Orthop. 2011 Oct;82(5):610-3.	Patients < 60 years
Lattmann et al 2008	Comparison of 2 surgical approaches for volar locking plate osteosynthesis of the distal radius. J Hand Surg Am. 2008 Sep;33(7):1135-43.	Other comparison
Löw et al 2020	The Requirement for Closed Reduction of Dorsally Displaced Unstable Distal Radius Fractures Before Operative Treatment. Dtsch Arztebl Int. 2020 Nov 13;117(46):783-789.	Other comparison
Lutz et al 2014	Complications associated with operative versus nonsurgical treatment of distal radius fractures in patients aged 65 years and older. J Hand Surg Am. 2014 Jul;39(7):1280-6.	Nonrandomized controlled trial
Ma et al 2016	External fixation is more suitable for intra-articular fractures of the distal radius fracture in elderly patients. Bone Res. 2014; 4: 16017.	Nonrandomized controlled trial
Manrique et al 2017	Percutaneous pinning vs. internal fixation with locking plate: Postoperative results of comminuted fractures of the distal radius metaphysis. A randomised controlled trial. Rev Colomb Ortop Traumatol. 2017; 31(3): 114-9.	Patients < 60 years
Mardani et al 2011	Distal Radius Fracture, a Comparison Between Closed Reduction and Long Arm Cast Vs. Closed Reduction and Percutaneous Pinning and Short Arm Cast. Shiraz E-Medical Journal. 2011; 12(3): 151-61.	Patients < 60 years
McFayden 2011	Should unstable extra-articular distal radial fractures be treated with fixed-angle volar-locked plates or percutaneous Kirschner wires? A prospective randomised controlled trial. Injury. 2011 Feb;42(2):162-6.	Patients < 60 years
McQueen et al 1996	Redisplaced unstable fractures of the distal radius: a prospective randomised comparison of four methods of treatment. J Bone Joint Surg Br. 1996 May;78(3):404-9.	Patients < 60 years
McQueen et al 1998	Redisplaced unstable fractures of the distal radius. A randomised, prospective study of bridging versus non-bridging external fixation. J Bone Joint Surg Br. 1998 Jul;80(4):665-9.	Patients < 60 years

Mirhamidi et al 2013	A prospective comparison between Kapandji and percutaneous extra-focal fixation in extra articular distal radius fractures. Int J Clin Exp Med. 2013;6(2):133-9.	Patients < 60 years
Mishra et al 2021	A comparative study of variable angle volar plate and bridging external fixator with K-wire augmentation in comminuted distal radius fractures. Chin J Traumatol. 2021 Sep;24(5):301-305.	Patients < 60 years
Mulders et al 2019	Volar Plate Fixation Versus Plaster Immobilization in Acceptably Reduced Extra-Articular Distal Radial Fractures: A Multicenter Randomized Controlled Trial. J Bone Joint Surg Am. 2019 May 1;101(9):787-796.	Patients < 60 years
Nishiwaki et al 2021	A Prospective Randomized Comparison of Variable-Angle and Fixed-Angle Volar Locking Plating for Intra-Articular Distal Radius Fractures. J Hand Surg Am. 2021 Jul;46(7):584-593.	Patients < 60 years
Oshige et al 2007	A comparative study of clinical and radiological outcomes of dorsally angulated unstable distal radius fractures in elderly patients: intrafocal pinning versus volar locking plating. J Hand Surg Am. 2007 Nov;32(9):1385-92.	Nonrandomized controlled trial
Plate et al 2015	Randomized comparison of volar locking plates and intramedullary nails for unstable distal radius fractures. J Hand Surg Am. 2015 Jun;40(6):1095-101.	Patients < 60 years
Pritchett 1995	External fixation or closed medullary pinning for unstable Colles fractures? J Bone Joint Surg Br. 1995 Mar;77(2):267-9.	Patients < 60 years
Rahman et al 2012	Treatment of unstable intraarticular fracture of distal radius: POP casting with external fixation. J Pak Med Assoc. 2012 Apr;62(4):358-62.	Patients < 60 years
Roh et al 2015	A randomized comparison of volar plate and external fixation for intra-articular distal radius fractures. J Hand Surg Am. 2015 Jan;40(1):34-41.	Patients < 60 years
Roumen et al 1991	Unstable Colles' fractures in elderly patients. A randomised trial of external fixation for redisplacement. J Bone Joint Surg Br. 1991 Mar;73(2):307-11.	Patients < 60 years
Rozental et al 2009	Functional outcomes for unstable distal radial fractures treated with open reduction and internal fixation or closed reduction and percutaneous fixation. A prospective randomized trial. J Bone Joint Surg Am. 2009 Aug;91(8):1837-46.	Patients < 60 years
Safi et al 2013	Treatment of extra-articular and simple articular distal radial fractures with intramedullary nail versus volar locking plate. J Hand Surg Eur Vol. 2013 Sep;38(7):774-9.	Patients < 60 years
Schmelzer-Schmied et al 2009	Comparison of external fixation, locking and non-locking palmar plating for unstable distal	Nonrandomized controlled trial

	radius fractures in the elderly. Int Orthop. 2009 Jun;33(3):773-8.	
Schønnemann et al 2011	Randomised study of non-bridging external fixation compared with intramedullary fixation of unstable distal radial fractures. J Plast Surg Hand Surg. 2011 Sep;45(4-5):232-7.	Patients < 60 years
Sharma et al 2014	Outcomes and complications of fractures of distal radius (AO type B and C): volar plating versus nonoperative treatment. J Orthop Sci. 2014 Jul;19(4):537-44.	Patients < 60 years
Sharma et al 2020	Prospective Randomized Study Comparing the External Fixator and Volar Locking Plate in Intraarticular Distal Radius Fractures: Which Is Better? Cureus. 2020 Feb 2;12(2):e6849.	Patients < 60 years
Shukla et al 2014	External fixation versus volar locking plate for displaced intra-articular distal radius fractures: a prospective randomized comparative study of the functional outcomes. J Orthop Traumatol. 2014 Dec;15(4):265-70.	Patients < 60 years
Sirniö et al 2019	Early palmar plate fixation of distal radius fractures may benefit patients aged 50 years or older: a randomized trial comparing 2 different treatment protocols. Acta Orthop. 2019 Apr;90(2):123-128.	Patients < 60 years
Subramanian et al 2015	“A Comparative Study between “Closed Reduction and Cast Immobilisation “And” Closed Reduction, Percutaneous K-Wire Fixation, Cast Immobilisation” In Distal Radius Fractures”. Journal of Evolution of Medical and Dental Sciences 2015; 67(4): 11714-23.	Patients < 60 years
Toon et al 2017	Outcomes and financial implications of intra-articular distal radius fractures: a comparative study of open reduction internal fixation (ORIF) with volar locking plates versus nonoperative management. J Orthop Traumatol. 2017 Sep;18(3):229-234.	Patients < 60 years
Uchikura et al 2004	Comparative study of nonbridging and bridging external fixators for unstable distal radius fractures. J Orthop Sci. 2004;9(6):560-5.	Patients < 60 years
Venkatesh et al 2016	Comparative Study between Closed Reduction and Cast Application Versus Percutaneous K-Wire Fixation for Extra-Articular Fracture Distal end of Radius. J Clin Diagn Res. 2016 Feb;10(2):RC05-9.	Patients < 60 years
Wei et al 2009	Unstable distal radial fractures treated with external fixation, a radial column plate, or a volar plate. A prospective randomized trial. J Bone Joint Surg Am. 2009 Jul;91(7):1568-77.	Patients < 60 years
Wilke et al 2011	Wrist function recovers more rapidly after volar locked plating than after external fixation but the outcomes are similar after 1 year. Acta Orthop. 2011 Feb;82(1):76-81.	Patients < 60 years
Williksen et al 2013	Volar locking plates versus external fixation and adjuvant pin fixation in unstable distal radius	Patients < 60 years

	fractures: a randomized, controlled study. J Hand Surg Am. 2013 Aug;38(8):1469-76.	
Williksen et al 2015	External Fixation and Adjuvant Pins Versus Volar Locking Plate Fixation in Unstable Distal Radius Fractures: A Randomized, Controlled Study With a 5-Year Follow-Up. J Hand Surg Am. 2015 Jul;40(7):1333-40.	Patients < 60 years
Xu et al 2009	Prospective randomised study of intra-articular fractures of the distal radius: comparison between external fixation and plate fixation. Ann Acad Med Singap. 2009 Jul;38(7):600-6.	Patients < 60 years
Young et al 2003	Seven-year outcome following Colles' type distal radial fracture. A comparison of two treatment methods. J Hand Surg Br. 2003 Oct;28(5):422-6.	Patients < 60 years
Zehir et al 2014	Intramedullary repair device against volar plating in the reconstruction of extra-articular and simple articular distal radius fractures; a randomized pilot study. Int Orthop. 2014 Aug;38(8):1655-60.	Patients < 60 years
