# Supplementary material for: Effectiveness and Safety of Different Treatment Modalities for Patients Older Than 60 Years with Distal Radius Fracture: A Network Meta-Analysis of Clinical Trials
Source: Int J Environ Res Public Health. 2023 Feb 19;20(4):3697. doi: 10.3390/ijerph20043697 (PMC9965012; doi:10.3390/ijerph20043697)
Supplement: Supplementary file 1 [file ijerph-20-03697-s001.zip › Table S4. Characteristics studies.pdf]

Table S4. Characteristics of the randomized controlled trials included in this network meta-analyses.

STUDY		PATIENTS					TREATMENT MODALITIES				OUTCOMES
**Author/ year**	**Country**	**Patients**	**Age (y) Mean ± SD**	**Female Number (%)**	**Dominant side involved Number (%)**	**Type of DRF**	**Groups**	**Description of intervention**	**Time of surgery Mean ± SD**	**Time of immobilization post treatment**	**Outcomes / Follow-up**
**Arora et al 2011**	Austria	36	75.9 ± NR	28 (77.8)	15 (41.7)	Unstable intra or extra-articular DRF (AO type: A2 3; A3 7; C1 4; C2 12; C3 10)	IG 1: VLP	Open reduction and internal fixation with VLP	NR	After surgery, the wrist was immobilized for 10 days, then the sutures were removed and the wrist was placed in a removable splint for 1 week	6, 12 weeks, 6 months, and 1 year follow-up<br><br>Wrist ROM:<br>-Flexion<br>-Extension<br>-Pronation<br>-Supination<br>-Radial deviation<br>-Ulnar deviation<br><br>Grip strength<br><br>VAS<br><br>DASH<br><br>PRWE<br><br>Complications
		37	77.4 ± NR	27 (73)	21 (56.8)	Unstable intra or extra-articular DRF (AO type: A2 3; A3 9; C1 11; C2 8; C3 6)	CG: Non operative treatment	Closed reduction and cast immobilization		5 weeks	
**Atroshi et al 2006**	Sweden	19	71 ± NR	15 (78.9)	10 (52.6)	Unstable intra or extra-articular DRF (AO type: A2/A3 8; C2/C3 11)	IG 1: BEF	The Hoffmann external fixator was used. No additional fixation was used	27 ± 11 min	After surgery the wrist was immobilized for 6 weeks	10 weeks, 6 months, and 1 year follow-up<br><br>Wrist ROM:<br>-Flexion<br>-Extension<br>-Pronation
		19	70 ± NR	16 (84.2)	9 (47.4)	Unstable intra or extra-articular DRF (AO type:	IG 2: NBEF	The Hoffmann Compact external fixation	37 ± 11 min	After surgery the wrist was immobilized for 6 weeks	

						A2/A3 11; C2/C3 8)		was used. No additional fixation was used			-Supination<br>-Radial deviation<br>-Ulnar deviation<br><br>Grip strength<br><br>VAS<br><br>DASH<br><br>SF-12<br><br>Complications
**Azzopardi et al 2005**	Scotland	27	72 ± 8	23 (85.2)	10 (37)	Unstable, dorsally angulated and extraarticular DRF (A3 type AO)	IG 1: PKW	Closed reduction, supplementary percutaneous pinning (k-wires), and cast immobilization	NR	After surgery the wrist was immobilized for 5 weeks, then wires were removed	1-year follow-up<br><br>Wrist ROM:<br>-Flexion<br>-Extension<br>-Pronation<br>-Supination<br>-Radial deviation<br>-Ulnar deviation<br><br>Grip strength<br><br>ADL<br><br>VAS<br><br>SF-36<br><br>Complications
		27	71 ± 9	25 (92.6)	12 (44.4)	Unstable, dorsally angulated and extraarticular DRF (A3 type AO)	CG: Non operative treatment	Closed reduction and cast immobilization		5 weeks	
**Bartl et al 2014**	Germany	86	75.3 ± 6.7	77 (90)	76 (89)	Unstable intraarticular DRF (AO type: C1 36; C2 35; C3 15)	IG 1: VLP	Open reduction and internal fixation with VLP	NR	2 weeks	3 months, and 1 year follow-up<br><br>Wrist ROM:<br>-Flexion<br>-Extension<br>-Pronation<br>-Supination
		88	74.4 ± 7.1	76 (86)	84 (97)	Unstable intraarticular DRF (AO type: C1	CG: Non operative treatment	Closed reduction and cast immobilization		6 weeks	

						40; C2 35; C3 13)					-Radial deviation<br>-Ulnar deviation<br><br>SF-36<br><br>DASH<br><br>EQ-VAS<br><br>EQ-5D index<br><br>Complications
		35	75.8 ± 9.3	30 (85.7)	NR	Unstable intra or extra-articular DRF (AO type: A 16; C 19)	CG: Non operative treatment	Closed reduction and cast immobilization		6 weeks	
		15	71.3 ± 13.6	13 (86.7)	7 (46.7)	Dorsally displaced extra-articular DRF (AO type: A2 10; A3 5)	IG 2: VLP	Open reduction and internal fixation with VLP	NR	NR	
**Chung et al 2020; Chung et al 2021**	Multicenter (Canada, Singapore, and United States)	65	67 ± 6.2	55 (84.6)	NR	Unstable intra or extra-articular DRF (AO type: A2 33; A3 4; C1 3; C2 20; C3 1)	IG 1: VLP	Open reduction and internal fixation with VLP	68 ± 34.3 min	NR	6 weeks, 1- and 2-year follow-up.<br><br>MHQ<br><br>SF-36<br><br>Grip strength<br><br>Pinch strength<br><br>Wrist ROM:<br>-Flexion<br>-Extension<br>-Pronation<br>-Supination<br>-Radial deviation<br>-Ulnar deviation<br><br>Complications
		64	70 ± 8.4	59 (92.2)	NR	Unstable intra or extra-articular DRF (AO type: A1 1; A2 26; A3 9; C1 3; C2 20; C3 1)	IG 2: BEF	Closed reduction and bridging EF with or without supplemental k-wires	54 ± 23.3 min	NR	
		58	68 ± 7	49 (84.5)	NR	Unstable intra or extra-articular DRF (AO type: A2 30; A3 7; C1 3; C2 16)	IG 3: PKW	Closed reduction, supplementary percutaneous k-wires fixation, and immobilization	41 ± 38.5 min	NR	
		109	76 ± 10	93 (85.3)	NR	Unstable intra or extra-articular DRF (AO type: A1 1; A2 40; A3 12; C1 10; C2	GC: Non operative treatment	Closed reduction and cast immobilization		6 weeks	

						25; C3 2)					
**Foldhazy et al 2010**	Sweden	28	73.5 ± NR	24 (85.7)	17 (60.7)	Unstable intra or extra-articular DRF (AO type: A2 1; A3 10; C2 7; C3 4)	IG 1: BEF	Closed reduction, BEF without supplemental k-wires, and immobilization	NR	5 weeks	2, 6 months, and 1 year follow-up.<br><br>GOBC<br><br>VAS<br><br>Grip strength<br><br>Wrist ROM:<br>-Flexion<br>-Extension<br>-Pronation<br>-Supination<br>-Radial deviation<br>-Ulnar deviation<br><br>Complications
		31	71.5 ± NR	29 (93.6)	12 (38.7)	Unstable intra or extra-articular DRF (AO type: A2 5; A3 11; C1 1; C2 8; C3 4)	GC: Non operative treatment	Closed reduction and cast immobilization		5 weeks	
**Goehre et al 2014**	Germany	21	71.3 ± 5.7	18 (85.7)	NR	Unstable intra or extra-articular DRF (AO type: A2 4; A3 14; C1 3)	IG 1: VLP	Open reduction and internal fixation with VLP	60 min ± NR	1 week	3, 6 months, and 1 year follow-up.<br><br>DASH<br><br>PRWE<br><br>Castain Score<br><br>Grip strength<br><br>Wrist ROM:<br>-Flexion<br>-Extension<br>-Pronation<br>-Supination<br>-Radial deviation<br>-Ulnar deviation
		19	73.8 ± 8.9	19 (100)	NR	Unstable intra or extra-articular DRF (AO type: A2 9; A3 6; C1 4)	IG 2: PKW	Closed reduction, supplementary percutaneous k-wires fixation (combined Kapandji and Willenegger techniques), and immobilization	23 min ± NR	6 weeks	

											Complications
**Hasselllund et al 2021**	Norwegian	50	73.4 ± NR	47 (94)	24 (48)	Unstable intra or extra-articular DRF (AO type: A2 3; A3 9; C1 13; C2 16; C3 9)	IG 1: VLP	Open reduction and internal fixation with VLP	NR	2 weeks	3, 6 months, and 1 year follow-up<br><br>Quick DASH<br><br>PRWE<br><br>EQ-5D index<br><br>EQ-VAS<br><br>Grip strength<br><br>Wrist ROM:<br>-Flexion<br>-Extension<br>-Pronation<br>-Supination<br>-Radial deviation<br>-Ulnar deviation<br><br>Complications
		50	73.9 ± NR	42 (84)	18 (36)	Unstable intra or extra-articular DRF (AO type: A2 2; A3 12; C1 11; C2 18; C3 7)	CG: Non operative treatment	Closed reduction and cast immobilization		5.5 weeks	
**Hegeman et al 2004**	Netherland	15	71 ± 7.9	13 (86.7)	NR	Unstable intra-articular DRF (AO type: C2 9; C3 6)	IG 1: BEF	Closed reduction, Hoffmann II Compact external fixation, and immobilization	NR	6 weeks	6 weeks, 3 months, and 1 year follow-up<br><br>Gartland and Werley Score<br><br>Grip strength<br><br>Wrist ROM:<br>-Flexion<br>-Extension<br><br>Complications
		17	69 ± 8	16 (94.1)	NR	Unstable intra-articular DRF (AO type: C2 14; C3 3)	CG: Non operative treatment	Closed reduction and cast immobilization		6 weeks	
		20	66 ± NR	25 (100)	6 (24)	Dorsally displaced intra	IG 1: PKW	Closed reduction,	32 ± 14 min	5 weeks	3 months, and 1

**Hollevoet et al 2011**	Belgium					or extra-articular DRF (intra articular 13)		supplementary percutaneous pinning (k-wires), and cast immobilization			year follow-up<br><br>Wrist ROM:<br>-Flexion-extension arc<br>-Pronation – supination arc<br><br>Grip strength<br><br>DASH<br><br>Complications
		20	67 ± NR	16 (80)	9 (45)	Dorsally displaced intra or extra-articular DRF (intra articular 9)	IG 2: VLP	Open reduction and internal fixation with VLP	65 ± 20 min	5 weeks	
**Horne et al 1990**	New Zealand	14	70.6 ± 5.4	NR	NR	Displaced intra or extra-articular DRF (Frykman type: extra articular 10; intra articular 5)	IG 1: BEF	Closed reduction, BEF without supplemental k-wires, and immobilization	NR	5 weeks	4 to 15 months follow-up<br><br>Functional assessment score
		15	73.9 ± 8.6	NR	NR	Displaced intra or extra-articular DRF (Frykman type: extra articular 6; intra articular 8)	CG: Non operative treatment	Closed reduction and cast immobilization		5 weeks	
**Jakubietz et al 2008; Jakubietz et al 2012**	Switzerland	15	64.5 ± NR	13 (86.7)	15 (100)	Unstable intra-articular DRF (AO type: C1 10; C2 4; C3 1)	IG 1: VLP	Open reduction and internal fixation with VLP	NR	2 weeks	6 weeks, 3, 6 months, and 1 year follow-up<br><br>Wrist ROM:<br>-Flexion-extension arc<br>-Pronation – supination arc<br>-Radial – Ulnar arc<br><br>Grip strength
		15	66.3 ± NR	12 (80)	10 (66.7)	Unstable intra-articular DRF (AO type: C1 10; C2 3; C3 2)	IG 2: DPF	Open reduction and internal fixation with DPF	NR	2 weeks	

											VAS<br><br>Gartland Werley Score<br><br>Complications
**Jeudy et al 2012**	France	39	64.6 ± 3.5	31 (79.5)	17 (43.6)	Unstable intra-articular DRF (AO type: C2 34; C3 5)	IG 1: BEF	Closed reduction, Hoffmann II external fixation, with supplemental k-wires, and immobilization	NR	6 weeks	6 weeks, 3- and 6-months follow-up<br><br>Wrist ROM:<br>-Flexion-extension arc<br><br>Grip strength<br><br>GOBC<br><br>PRWE<br><br>Complications
		46	64.7 ± 3.7	26 (56.5)	18 (38.9)	Unstable intra-articular DRF (AO type: C2 26; C3 10)	IG 2: VLP	Open reduction and internal fixation with VLP	NR	6 weeks	
**Koshimune et al 2005**	Japan	22	68 ± NR	18 (81.8)	NR	Unstable intra or extra-articular DRF (AO type: A2 8; A3 1; C1 7; C2 6)	IG 1: VLP	Open reduction and internal fixation with VLP	NR	2 weeks	6 to 24 months follow-up<br><br>Wrist ROM:<br>-Flexion<br>-Extension<br>-Pronation<br>-Supination<br><br>Gartland Werley Score<br><br>Complications
		31	74 ± NR	28 (90.3)	NR	Unstable intra or extra-articular DRF (AO type: A2 10; A3 2; C1 5; C2 14)	IG 2: VNLP	Open reduction and internal fixation with VNLP	NR	2 weeks	
**Lawson et al 2021**	Australia and New Zealand	81	70.5 ± 7	70 (86.4)	NR	Unstable intra or extra-articular DRF (AO type: A 55; C 26)	IG 1: VLP	Open reduction and internal fixation with VLP	NR	2 weeks	3 months, and 1 year follow-up<br><br>PRWE<br><br>DASH<br><br>EQ-5D index<br><br>EQ-VAS
		85	71.3 ± 7.6	75 (88.2)	NR	Unstable intra or extra-articular DRF	CG: Non operative	Closed reduction and cast immobilization		6 weeks	

						(AO type: A 49; C 35)	treatment				NRS<br><br>Complications
**Marcheix et al 2010**	France	53	73 ± 11	48 (90.6)	32 (60.4)	Unstable intra or extra-articular DRF (AO type: A2 1; A3 22; C2 23; C3 6)	IG 1: PKW	Closed reduction, supplementary percutaneous pinning (k-wires), and cast immobilization	NR	6 weeks	3- and 6-months follow-up<br><br>Wrist ROM:<br>-Flexion<br>-Extension<br>-Pronation<br>-Supination<br><br>DASH<br><br>Grip strength<br><br>Herzberg Score<br><br>Complications
		50	75 ± 11	38 (76)	22 (44)	Unstable intra or extra-articular DRF (AO type: A3 17; C2 25; C3 8)	IG 2: VLP	Open reduction and internal fixation with VLP	NR	3 weeks	
**Martínez-Méndez et al 2017**	Spain	50	67 ± 8	39 (78)	35 (70)	Unstable intra-articular DRF (AO type: C1 23; C2 23; C3 4)	IG 1: VLP	Open reduction and internal fixation with VLP	NR	1 week	2 years follow-up<br><br>Wrist ROM:<br>-Flexion<br>-Extension<br>-Pronation<br>-Supination<br><br>Grip strength<br><br>VAS<br><br>DASH<br><br>PRWE<br><br>Complications
		47	70 ± 7	37 (78.7)	30 (63.8)	Unstable intra-articular DRF (AO type: C1 22; C2 20; C3 5)	CG: Non operative treatment	Closed reduction and cast immobilization	NR	6 weeks	
		70	63 ± NR	63 (90)	34 (48.6)	Unstable intra or extra-articular DRF (AO type: A2 5; A3 25; C1	IG 1: VLP	Open reduction and internal fixation with VLP	NR	4 weeks	6 weeks, 3 months, 1- and 3-years follow-up<br><br>Wrist ROM:

**Mellstrand-Navarro et al 2016; Saving et al 2019a**	Sweden					35; C2 3; C3 2)					-Flexion<br>-Extension<br>-Pronation<br>-Supination<br>-Radial deviation<br>-Ulnar deviation<br><br>DASH<br><br>Grip strength<br><br>PRWE<br><br>EQ-5D<br><br>Complications
		69	63 ± NR	64 (92.8)	28 (40.6)	Unstable intra or extra-articular DRF (AO type: A2 5; A3 20; C1 38; C2 4; C3 2)	IG 2: BEF	Closed reduction, Hoffmann Compact external fixation with supplemental k-wires, and immobilization	NR	5.5 weeks	
**Moroni et al 2004**	Italy	20	>65	NR	NR	Unstable extra-articular DRF (AO type: A2/A3)	IG 1: BEF	Closed reduction, Orthofix Pennig II external fixation with supplemental k-wires, and immobilization	NR	6 weeks	3 months follow-up<br><br>Horesh Demerit Point Wrist<br><br>SF-36<br><br>Complications
		20	>65	NR	NR	Unstable extra-articular DRF (AO type: A2/A3)	CG: Non operative treatment	Closed reduction and cast immobilization	NR	6 weeks	
**Saving et al 2019**	Sweden	58	80 ± NR	55 (94.8)	27 (46.6)	Unstable intra or extra-articular DRF (AO type: A2 5; A3 25; C1 35; C2 3; C3 2)	IG 1: VLP	Open reduction and internal fixation with VLP	NR	2 weeks	3 months, and 1 year follow-up<br><br>PRWE<br><br>DASH<br><br>EQ-5D<br><br>Wrist ROM:<br>-Flexion<br>-Extension<br>-Pronation
		64	78 ± NR	56 (87.5)	23 (35.9)	Unstable intra or extra-articular DRF (AO type: A2 5; A3 25; C1 35; C2 3; C3 2)	CG: Non operative treatment	Closed reduction and cast immobilization		4.5 weeks	

											-Supination<br>-Radial deviation<br>-Ulnar deviation<br><br>Grip strength
**Tahir et al 2021**	Pakistan	87	81 ± 3	16 (18.4)	60 (69)	Unstable intra or extra-articular DRF (AO type: A2 31; A3 28; C1 9; C2 9; C3 10)	IG 1: VLP	Open reduction and internal fixation with VLP	NR	NR	3 months, and 1 year follow-up<br><br>PRWE<br><br>Quick DASH<br><br>Mayo Wrist Score<br><br>SF-12<br><br>Wrist ROM:<br>-Flexion<br>-Extension<br>-Pronation<br>-Supination<br><br>Grip strength
		72	81 ± 2	17 (23.6)	45 (62.5)	Unstable intra or extra-articular DRF (AO type: A2 38; A3 3; C1 18; C2 9; C3 4)	CG: Non operative treatment	Closed reduction and cast immobilization		4.5 weeks	
**Wong et al 2010**	China	30	70 ± NR	24 (80)	21 (70)	Unstable extra-articular DRF (Frykman type: I 17; II 13)	IG 1: PKW	Closed reduction, supplementary percutaneous pinning (k-wires), and cast immobilization	NR	6 weeks	1 year follow-up<br><br>Wrist range of motion:<br>-Flexion<br>-Extension<br>-Pronation<br>-Supination<br>-Radial deviation<br>-Ulnar deviation<br><br>Grip Strength
		30	71 ± NR	25 (83.3)	20 (66.7)	Unstable extra-articular DRF (Frykman type: I 18; II 12)	CG: Non operative treatment	Closed reduction and cast immobilization		6 weeks	

											Mayo Wrist Score<br><br>WHOQoL<br><br>Complications

ADL: Activities of Daily Living; **BEF:** Bridging External Fixation; **CG:** Control Group; **DASH:** Disabilities of the Arm, Shoulder and Hand questionnaire; **DPF:** Dorsal Plate Fixation; **DRF:** Distal Radius Fracture; **EQ-5D** index: EuroQol 5D questionnaire; **EQ-VAS:** EuroQol Visual Analog Scale; **GOBC:** Green & O´Brian/Coney questionnaire; **IG:** Intervention Group; **IMN:** Intramedullary Nail; **MHQ**; Michigan Hand Questionnaire; **NBEF:** Non Bridging External Fixation; **NR:** Not Reported; **NRS**; Numerical Rating Scale; **PKW:** Percutaneous K-wire; **PRWE:** Patient Rated-Wrist Evaluation questionnaire; **ROM:** Range of Motion; **SD:** Standard Deviation; **SF-12:** 12-Item Short Form Survey of Health; **SF-36:** 36-Item Short Form Survey of Health; **VAS:** Visual Analog Scale; **VLP:** Volar Locking Plate; **VNLP:** Volar Non-Locking Plate; **WHOQoL:** World Health Organization Quality of Life questionnaire.
