# Supplementary material for: Effectiveness and Safety of Different Treatment Modalities for Patients Older Than 60 Years with Distal Radius Fracture: A Network Meta-Analysis of Clinical Trials
Source: Int J Environ Res Public Health. 2023 Feb 19;20(4):3697. doi: 10.3390/ijerph20043697 (PMC9965012; doi:10.3390/ijerph20043697)
Supplement: Supplementary file 1 [file ijerph-20-03697-s001.zip › Table S5. Subgroup analyses of different treatment modalities on functional outcomes, by time of follow-up..pdf]

Table S5. Subgroup analyses of different treatment modalities on functional outcomes, by time of follow-up.

**Comparisons**	**Follow-up (months)**	**Number studies**	**Grip Strength MD (95% CI)**	**I^2 (%)**	**p-value**	**Number studies**	**DASH MD (95% CI)**	**I^2 (%)**	**p-value**	**Number studies**	**PRWE MD (95% CI)**	**I^2 (%)**	**p-value**
**VLP / CI**	3	6	**13.9 (4.2, 23.5)**	94.3	0.005	5	**-5.7 (-8.2, -3.1)**	0	0.000	5	**-9.2 (-18.1, -0.3)**	87.2	0.044
**VLP / CI**	12	6	**7.1 (1.1, 13.1)**	84.3	0.019	7	-0.6 (-2.1, 0.9)	57.6	0.467	5	-2.7 (-5.8, 0.5)	38	0.094
**VLP / CI**	24	2	6.7 (-1.3, 15.1)	0	0.1	1	**-12 (-19.1, -4.9)**	NA	0.001	1	**-13 (-20.9, -5.1)**	NA	0.001

**VLP / PKW**	3	4	**9.9 (3.9, 15.8)**	0	0.001	3	-4.7 (-10.4, 0.9)	0	0.103	NA	NA	NA	NA
**VLP / PKW**	12	3	-12 (-33.9, 9.9)	0	0.858	NA	NA	NA	NA	NA	NA	NA	NA

CI: Confidence interval; MD: Mean difference; NA: Not available; DASH: Disabilities of the Arm, Shoulder and Hand questionnaire; PRWE: Patient-Rated Wrist Evaluation questionnaire. MD **in bold**: statistically significant.
