# Supplementary material for: Effectiveness and Safety of Different Treatment Modalities for Patients Older Than 60 Years with Distal Radius Fracture: A Network Meta-Analysis of Clinical Trials
Source: Int J Environ Res Public Health. 2023 Feb 19;20(4):3697. doi: 10.3390/ijerph20043697 (PMC9965012; doi:10.3390/ijerph20043697)
Supplement: Supplementary file 1 [file ijerph-20-03697-s001.zip › Table S6. Subgroup analyses of different treatment modalities on clinical complications, by time of follow-up..pdf]

Table S6. Subgroup analyses of different treatment modalities on clinical complications, by time of follow-up.

Comparisons	Follow-up (months)	Number studies	Overall Complications RR (95% CI)	I^2 (%)	p-value	Number studies	Minor Complications RR (95% CI)	I^2 (%)	p-value	Number studies	Mayor complications RR (95% CI)	I^2 (%)	p-value
**VLP / CI**	12	7	0.79 (0.38, 1.66)	84.6	0.538	7	0.57 (0.24, 1.38)	79.9	0.214	8	1.35 (0.78, 2.32)	1.9	0.287
**VLP / CI**	24	1	**0.69 (0.34, 1.39)**	NA	0.012	1	**0.07 (0.01, 0.55)**	NA	0.011	NA	NA	NA	NA
**VLP / PKW**	6	1	**0.12 (0.02, 0.88)**	NA	0.037	1	**0.12 (0.02, 0.88)**	NA	0.037	NA	NA	NA	NA
**VLP / PKW**	12	2	0.61 (0.31, 1.21)	24.3	0.158	2	**0.37 (0.15, 0.95)**	0	0.039	2	18.38 (3.07, 110.04)	0	0.001
**VLP / BEF**	6	1	**0.5 (0.29, 0.86)**	NA	0.025	1	**0.5 (0.29, 0.86)**	NA	0.018	NA	NA	NA	NA
**VLP / BEF**	12	1	1.22 (0.91, 1.63)	NA	0.261	1	1.01 (0.67, 1.53)	NA	0.353	NA	NA	NA	NA
**PKW / CI**	12	2	1 (0.15, 6.85)	0	1.000	2	0.52 (0.04, 6.02)	0	0.597	2	6.36 (0.81, 49.99)	0	0.079

**BEF / CI**	3	1	0.11 (0.01, 1.99)	NA	0.136	1	0.11 (0.01, 1.99)	NA	0.136	NA	NA	NA	NA
**BEF / CI**	12	3	**3.42 (1.69, 6.95)**	0	0.001	4	3.42 (1.69, 6.95)	0	0.001	NA	NA	NA	NA

CI: Confidence interval; RR: Risk ratio; NA: Not available. MD **in bold**: statistically significant.
