# Supplementary material for: Effectiveness and Safety of Different Treatment Modalities for Patients Older Than 60 Years with Distal Radius Fracture: A Network Meta-Analysis of Clinical Trials
Source: Int J Environ Res Public Health. 2023 Feb 19;20(4):3697. doi: 10.3390/ijerph20043697 (PMC9965012; doi:10.3390/ijerph20043697)
Supplement: Supplementary file 1 [file ijerph-20-03697-s001.zip › Table S7. Subgroup analyses of different treatment modalities on functional outcomes, by type of DRF.pdf]

Table S7. Subgroup analyses of different treatment modalities on functional outcomes, by type of DRF.

Comparisons	Type of DRF	Number studies	Grip Strength MD (95% CI)	I^2 (%)	p-value	Number studies	DASH MD (95% CI)	I^2 (%)	p-value	Number studies	PRWE MD (95% CI)	I^2 (%)	p-value
**VLP / CI**	IA	1	9 (-2.9, 20.9)	NA	0.140	3	**-7.2 (-11.3, -2.9)**	22.3	0.001	1	**-13 (-20.9, -5.1)**	NA	0.001
**VLP / CI**	EA/IA	13	**9.8 (6, 13.6)**	90	0.000	10	**-2.3 (-4.2, -0.4)**	72	0.016	10	**-4.3 (-7.1, -1.5)**	78.8	0.003
**VLP / PKW**	EA/IA	7	**5.9 (1.3, 10.5)**	2.4	0.012	3	-4.7 (-10.4, 0.9)	0	0.103	NA	NA	NA	NA

EA: Extra-articular; IA: Intra-articular; CI: Confidence interval; MD: Mean difference; NA: Not available; DASH: Disabilities of the Arm, Shoulder and Hand questionnaire; PRWE: Patient-Rated Wrist Evaluation questionnaire. MD **in bold**: statistically significant.
