# Supplementary material for: Effectiveness and Safety of Different Treatment Modalities for Patients Older Than 60 Years with Distal Radius Fracture: A Network Meta-Analysis of Clinical Trials
Source: Int J Environ Res Public Health. 2023 Feb 19;20(4):3697. doi: 10.3390/ijerph20043697 (PMC9965012; doi:10.3390/ijerph20043697)
Supplement: Supplementary file 1 [file ijerph-20-03697-s001.zip › Table S8. Subgroup analyses of different treatment modalities on clinical complications, by type of DRF..pdf]

Table S8. Subgroup analyses of different treatment modalities on clinical complications, by type of DRF.

Comparisons	Type of DRF	Number studies	Overall Complications RR (95% CI)	I^2 (%)	p-value	Number studies	Minor Complications RR (95% CI)	I^2 (%)	p-value	Number studies	Mayor complications RR (95% CI)	I^2 (%)	p-value
**VLP / CI**	IA	2	**0.17 (0.09, 0.32)**	0	0.000	2	**0.07 (0.03, 0.18)**	0	0.000	2	2.58 (0.6, 11.12)	0	0.204
**VLP / CI**	EA/IA	6	1.05 (0.56, 1.96)	74.2	0.887	6	0.84 (0.42, 1.69)	63	0.627	6	1.25 (0.64, 2.43)	1.9	0.516
**VLP / PKW**	EA/IA	3	0.49 (0.21, 1.14)	24.3	0.098	3	**0.3 (0.13, 0.71)**	0	0.006	2	**18.38 (3.07, 110.04)**	0	0.001
**VLP / BEF**	IA	1	**0.5 (0.29, 0.86)**	NA	0.025	1	**0.5 (0.29, 0.86)**	NA	0.018	NA	NA	NA	NA
	EA/IA	1	1.22 (0.91, 1.63)	NA	0.261	1	1.01 (0.67, 1.53)	NA	0.353	NA	NA	NA	NA
**PKW / CI**	EA	2	1 (0.15, 6.85)	0	1.000	2	0.52 (0.04, 6.02)	0	0.597	2	6.36 (0.81, 49.99)	0	0.079
**BEF / CI**	EA	1	0.11 (0.01, 1.99)	NA	0.136	1	0.11 (0.01, 1.99)	NA	0.136	NA	NA	NA	NA
**BEF / CI**	IA	1	**3.12 (1.26, 7.74)**	NA	0.014	1	**3.12 (1.26, 7.74)**	NA	0.014	NA	NA	NA	NA
**BEF / CI**	EA/IA	2	**3.95 (1.28, 12.2)**	0	0.017	2	**3.95 (1.28, 12.2)**	0	0.017	NA	NA	NA	NA

EA: Extra-articular; IA: Intra-articular; CI: Confidence interval; RR: Risk ratio. MD **in bold**: statistically significant.
